# Early occurrence of acute myelomonocytic leukemia (M4/M5) after liver transplantation: a case report

**DOI:** 10.1186/s13256-023-04126-2

**Published:** 2023-09-05

**Authors:** Farhad Zamani, Hanie Karimi, Mohsenreza Mansoorian, Ali Basi, S. Ahmad Hosseini, Zahra Zahed, Nasim Seyedghasemipour, Roghayeh Sahraie

**Affiliations:** 1 Gastrointestinal and Liver Disease Research Center, Iran University of Medical Sciences, Tehran, Iran; 2 School of Medicine, Tehran University of Medical Sciences, Tehran, Iran; 3 Department of Surgery, Transplant Research Center, Iran University of Medical Sciences, Tehran, Iran; 4 Department of Hematology Oncology, Iran University of Medical Sciences, Tehran, Iran; 5 School of Medicine, Iran University of Medical Sciences, Tehran, Iran; 6 Department of Medical Sciences, Ardabil University of Medical Sciences, Ardabil, Iran; 7 Faculty of Medicine, Iran University of Medical Sciences, Tehran, Iran

**Keywords:** Acute myeloid leukemia, Liver transplantation, Acute myelomonocytic leukemia

## Abstract

**Introduction:**

Acute myeloid leukemia is a rare event in post-liver-transplantation recipients. In the present report, we described a case of extramedullary acute myeloid leukemia, M_4_/M_5_ subtype, following orthotopic liver transplant.

**Case presentation:**

The patient was a 50-year-old Iranian woman who underwent orthotopic liver transplant due to hepatitis B-related cirrhosis (Child C, MELD (model for end-stage liver disease score) = 22). Orthotopic liver transplant was performed using the piggy back technique in January 2022. Induction immunosuppressive therapy was 1 gm methylprednisolone for 3 days followed by a triple maintenance immunosuppressive regimen including mycophenolate mofetil, prednisolone, and tacrolimus. About 5 months after orthotopic liver transplant in June 2022, the patient presented with leukocytosis, with white blood cell count of 99.4 × 103/µl, and physical examination revealed only cervical lymphadenopathy. Biopsy of cervical lymph nodes showed a myeloid tumor. She was immediately hospitalized. Eight hours after hospitalization, the patient gradually developed lethargy and decreased O_2_ saturation to approximately 89%. Flow cytometry demonstrated the markers of a myelomonocytic acute myeloid leukemia (M_4_/M_5_). Cytoreduction was immediately started by intensive leukopheresis followed by induction therapy. Because of a septic complication during the induction therapy, further chemotherapy was discontinued and broad-spectrum antibiotics and antifungal treatments started. Unfortunately, our patient died of severe septic shock 42 days after hospitalization.

**Conclusion:**

Acute myeloid leukemia is a rare phenomenon after liver transplantation, and it can follow a rapidly fatal clinical course.

## Introduction

Liver transplantation is the main treatment for patients with acute liver failure and liver cirrhosis due to various causes [1]. Despite the benefits of transplantation, it can have some adverse effects mainly due to direct and indirect effects of immunosuppressive therapy [2]; for example, the incidence of de novo malignancies post-liver transplantation is up to 26% [3]. These malignancies occur as a result of immunosuppressive therapy being initiated for posttransplant recipients [4]. De novo malignancies in liver transplanted patients usually have an aggressive behavior [5]. In orthotopic liver transplant (OLT) recipients, Posttransplant lymphoproliferative disease (PTLD) and non-PTLD tumors generally occur in the early phases after liver transplantation, and their incidence is higher than in the general population [6, 7]. Acute myeloid leukemia (AML) is a rare phenomenon in liver transplant recipients, with a wide range of complications from septicemia, progressive disease, and relapse to early death, which is reported in 25% of cases [4]. In the present report, we described a case with AML occurrence after OLT.

## Case presentation

The patient was a 50-year-old Iranian woman with no familial and psychosocial history and no history of surgery or any interventions or any drug usage before. The only past medical history of the patient was hepatitis B-related cirrhosis with the Child C and model for end-stage liver disease (MELD) score = 22 (both scores are utilized to predict the cirrhotic patients outcomes: Child–Pugh is used in the assessment of liver dysfunction severity and MELD is used to rank the liver transplant candidates based on their priority [8]) who underwent OLT. OLT was performed using the piggyback technique in January 2022. Induction immunosuppressive therapy was methylprednisolone (1 g) for 3 days, followed by a triple maintenance immunosuppressive regimen including mycophenolate mofetil, prednisolone, and tacrolimus. Hepatitis B infection was successfully treated with the combination of hepatitis B immunoglobulin (HBIG) and tenofovir disoproxil fumarate.

After OLT, she was followed-up regularly according to our center’s standard posttransplant protocol. During follow-up, allograft function was normal, and other laboratory findings such as blood cells and leukocyte counts (Table 1), as well as biochemical parameters such as liver enzymes, showed no abnormalities and were in the normal range. Graft sonography revealed no abnormalities either. The overall status of the patient was also reported to be good.

**Table 1:** Hemoglobin, white blood cell, and platelet count in the patient after liver transplantation

Time after transplantation	Hemoglobin (g/dl)	WBC (×10^3^/µl)	Platelet (×10^3^/µl)
Zero	10.7	4900	357,000
1 week	11	5000	385,000
2 weeks	10.4	5100	300,000
3 weeks	9.9	4600	314,000
5 weeks	11.2	3900	282,000
7 weeks	11	4050	310,000
9 weeks	11.1	4600	360,000
13 weeks	10.9	4520	343,000

About 5 months after OLT, the patient presented with leukocytosis (WBC: 99.4 × 103/µl).

Up to this time, she was asymptomatic, and on physical examination, only cervical lymphadenopathy was notable; for further investigations, she was referred to an infectious disease specialist. She was tested for Epstein–Barr virus (EBV) serology, which was negative and underwent ultrasonography that showed multiple oval form lymphadenopathies in all neck zones, with the greatest one being the jugulodigastric node with short axis diameter (SAD) of 13 mm.

**Fig. 1:**
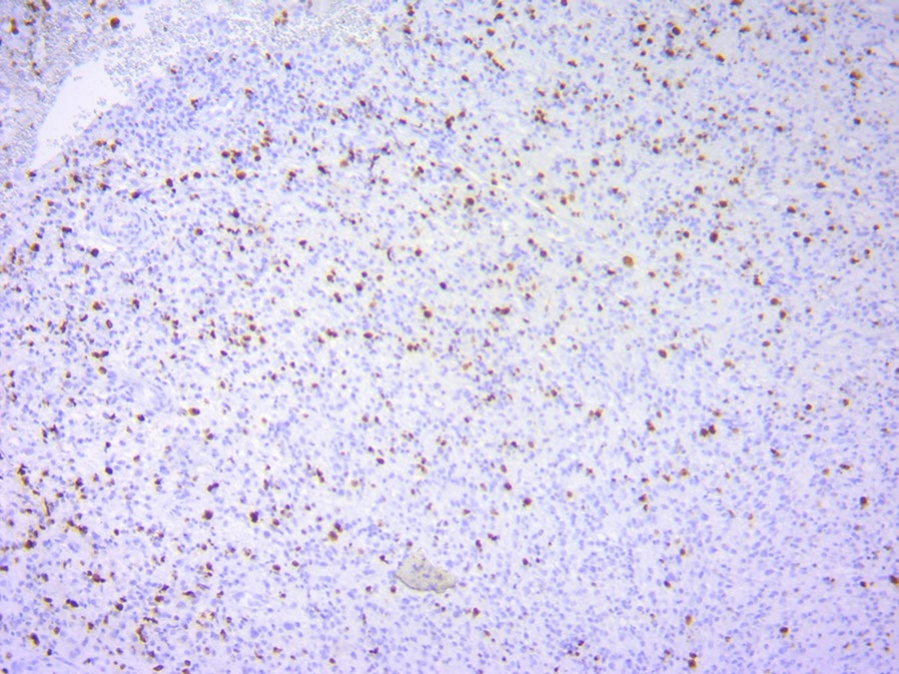
Lymph node histology

A guided biopsy was performed from cervical lymph nodes and showed hypercellular neoplastic tissue composed of diffuse infiltration of blastic cells with hyperchromic nuclei myeloid tumor (Fig. 1).

Hypercellular neoplastic tissue composed of diffuse infiltration of blastic cells with hyperchromic nuclei.

The repeated complete blood count (CBC) showed hyperleukocytosis (WBC: 200 × 10^3^/µl, Hb: 9.5 g/dl, platelet: 138 × 10^3^/µl); therefore, she was immediately hospitalized. After 8 h of hospitalization, the patient gradually developed lethargy and decreased O_2_ saturation to the SPO_2_ of 89%.

**Fig. 2:**
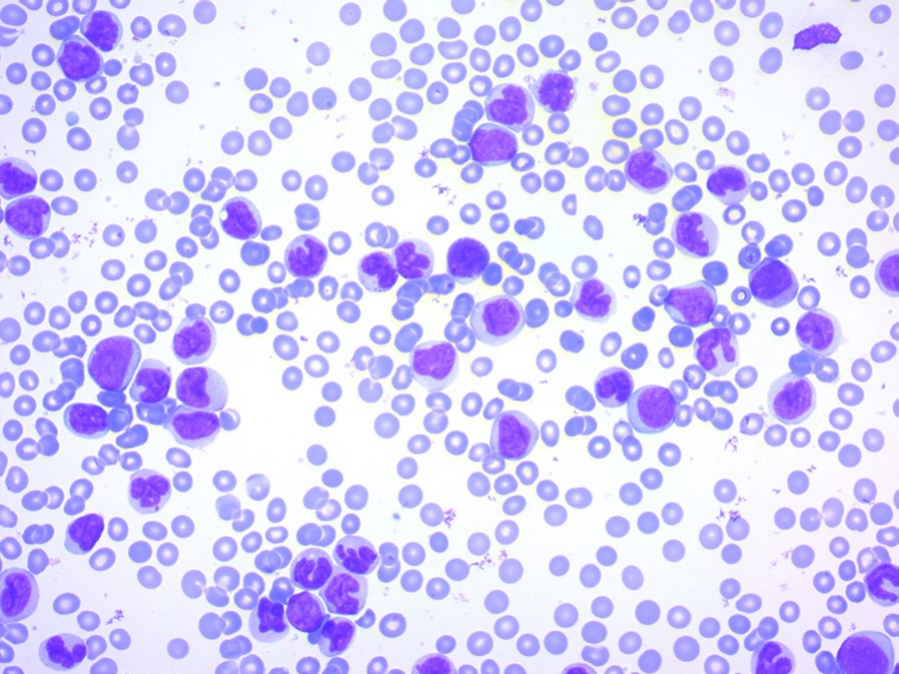
The peripheral blood smear showed many myeloid and monocytic cells, including monoblasts and promonocytes

On peripheral blood smear, there was leukocytosis and many medium-sized myelomonoblasts, hypochromia, anisocytosis, mild thrombocytopenia, a few smudge cells, and basket cells. (Fig. 2).

Flow cytometry demonstrated the markers of a myelomonocytic AML (HLA-DR, CD 13, CD 14, CD 33, CD 38, CD 45, CD 64). Double-positive (CD 13/CD 33) myeloid cells constituted 68% of blood mononuclear cells, and double-positive (CD 14/CD 64) monocytic cells were 65.7% (M_4_/M_5_) (Table 2). Due to the patient’s poor condition, bone marrow biopsy or aspiration (BMB/BMA) for cytogenetic analysis was not possible. Cytoreduction was immediately started by intensive leukopheresis followed by induction therapy. Because of a septic complication during the induction therapy, we discontinued further chemotherapy and started broad-spectrum antibiotics and antifungal treatments. Moreover, the doses of immunosuppressive medications were decreased. Finally, despite the mentioned efforts, a severe septic shock occurred, and the patient expired after 42 days of hospitalization.

**Table 2:** Flow cytometry immunophenotyping analysis

**HLA-DR**	94.9	**CD13**	68.6	Viability: about 80% Abnormal cells present by FCM:** Yes** Percentage of cells with abnormal phenotype:** About 80%** Cell size:** medium** Phenotype:** immature myelomonoid series**
**CD14**	66.4	CD20	0.9	
CD3	4.9	**CD33**	89.0	
CD19	0.8	CD34	16.0	
CD5	3.9	**CD38**	82.7	
*CD13/CD33*	68.0	**CD45**	97.9	
*CD14/CD64*	65.7	CD45 dim	92.3	
**CD64**	74.4			
DIAGNOSIS		**Non-M**_**3**_**-AML (M**_**4**_**/M**_**5**_**)**		

The bolded items are indicator markers of myelomonocytic AML. The Italic items are indicative of AML M4/M5 type

HLA-DR: human leukocyte antigen-DR isotype, CD: cluster of differentiation, FCM: flow cytometry

## Discussion

De novo malignancy is the second leading cause of mortality in liver-transplanted patients [9]. The incidence of malignancy after liver transplantation has been on the rise in the recent two decades (2000–2018) compared with earlier years (1987–1999) [10]. However, leukemia is an unusual malignancy following solid-organ transplantation. The relative risk of AML in heart and lung transplant recipients has been estimated as 5.5%, which is higher than that in other solid-organ transplantations such as kidney and liver (3).

In the current report, based on the patient’s history and the presence of cervical lymphadenopathy, as well as a short latency period (that is, about 5 months from transplantation), initially posttransplant lymphoproliferative disease (PTLD) was suspected. PTLD occurs in liver transplant recipients with immunosuppression and Epstein–Barr virus (EBV) plays a critical role in its pathogenesis. Up to 2.8% of liver transplant adult recipients may develop PTLD. Its risk factors include first-year posttransplant, EBV–seronegativity, age above 18 years, and intense immunosuppression [11, 12]. However, this was ruled out by cervical node biopsy and negative EBV serology, and AML occurrence was suspected. The development of fatigue, weakness, and cervical lymphadenopathy on physical examination, besides the evidence of hyperleukocytosis in CBC and the findings of flow cytometry, suggested AML (M_4_/M_5_ subtype). However, BMB and BMA were not possible for cytogenetic analysis and confirmation of diagnosis due to the patient’s unfavorable condition.

AML is characterized by the presence of > 20% blasts in the bone marrow or peripheral blood (6). However, for confirming the diagnosis and identifying the subtype, BMB and flow cytometry should be performed [13].

The first post-liver-transplantation AML (PT–AML) case was described by Thalhammer *et al*. in a 65-year-old man with hepatocellular carcinoma and cirrhosis in 1997 [4]. About 70% of PT–AML occurs within 5 years of transplantation, and most of them develop during the first 8 years [14].

Wu *et al*. reported several cases of myeloid neoplasms following solid-organ transplantation, including five cases of post-liver-transplantation AML with an interval period from transplantation to leukemia diagnosis of 12–156 months. Moreover, they showed that AML occurred earlier in liver-transplanted patients than in those who received other solid-organ transplants [15].

Posttransplant leukemia risk factors include immunosuppressive regimens, the genetic predisposition of the recipient and donor, advanced age, and viral infections. However, the role of background diseases such as primary sclerosing cholangitis (PSC), hepatitis B virus (HBV) infection and gender on the incidence of post-liver-transplantation AML is unclear [16–21].

Azathioprine as an immunosuppressive agent has been associated with de novo malignancy in transplanted patients, in a report of 19 patients with AML after transplantation, 16 had received azathioprine indicating its mutagenic properties and reinforcing the hypothesis that AML occurrence might be as a direct result of drug toxicity, unlike PTLD in which the viral infection following immunosuppressive condition is the main cause [4]. Also, high blood concentrations of tacrolimus (more than 20 ng/dl) have been noted to increase cancer risk [22]. In contrast, mycophenolate mofetil is known for its anticancer effects [23]. Our patient underwent a triple immunosuppressive regimen including prednisolone (20 mg), mycophenolate mofetil (II/bd), and tacrolimus (II/bd). She did not take azathioprine in the maintenance regimen.

The presence of hematopoietic stem cells in the liver suggests that post-liver-transplantation leukemia might be originated from the donor graft.

Bodo *et al*. demonstrated the development of donor-derived acute promyelocytic leukemia in a 57-year-old woman who underwent liver transplantation. In fact, HLA typing showed that leukemic cells in the recipient and donor were compatible together, confirming that blast cells came from the donor [24]. In another report, AML–M_4_ was diagnosed in the liver of a 43-year-old man who underwent liver transplantation, in whom tumor cells had similar HLA typing with the donor’s cells [25]. Unfortunately, we did not have access to the donor; therefore, we decided to follow a kidney recipient from the same donor. During 1 year after the transplantation, his examinations and laboratory tests, including CBC and other differentials, were normal, and no suspicious presentation was reported.

## Conclusion

Solid-organ transplants, especially liver transplants, need intense vigilance and surveillance in the early period after transplant due to the risk of de novo malignancies such as AML and their fatal consequences, although they are rare..

## Data Availability

The data are available with the corresponding author and can be reached on request.
